# *In situ* Linkage of Fungal and Bacterial Proliferation to Microbiologically Influenced Corrosion in B20 Biodiesel Storage Tanks

**DOI:** 10.3389/fmicb.2020.00167

**Published:** 2020-02-25

**Authors:** Blake W. Stamps, Caitlin L. Bojanowski, Carrie A. Drake, Heather S. Nunn, Pamela F. Lloyd, James G. Floyd, Katelyn A. Emmerich, Abby R. Neal, Wendy J. Crookes-Goodson, Bradley S. Stevenson

**Affiliations:** ^1^Department of Microbiology and Plant Biology, University of Oklahoma, Norman, OK, United States; ^2^UES, Inc., Dayton, OH, United States; ^3^711th Human Performance Wing, Airman Systems Directorate, Wright-Patterson AFB, Dayton, OH, United States; ^4^Soft Matter Materials Branch, Materials and Manufacturing Directorate, Air Force Research Laboratory, Wright-Patterson AFB, Dayton, OH, United States; ^5^Air Force Life Cycle Management Center, Mobility Directorate, Wright Patterson AFB, Dayton, OH, United States; ^6^Azimuth Corporation, Dayton, OH, United States

**Keywords:** biocorrosion, biofouling, fuel, biodiesel, B20, biodegradation

## Abstract

Renewable fuels hold great promise for the future yet their susceptibility to biodegradation and subsequent corrosion represents a challenge that needs to be directly assessed. Biodiesel is a renewable fuel that is widely used as a substitute or extender for petroleum diesel and is composed of a mixture of fatty acid methyl esters derived from plant or animal fats. Biodiesel can be blended up to 20% v/v with ultra-low sulfur diesel (i.e., B20) and used interchangeably with diesel engines and infrastructure. The addition of biodiesel, however, has been linked to increased susceptibility to biodegradation. Microorganisms proliferating via degradation of biodiesel blends have been linked to microbiologically influenced corrosion in the laboratory, but not measured directly in storage tanks (i.e., *in situ*). To measure *in situ* microbial proliferation, fuel degradation and microbially influenced corrosion, we conducted a yearlong study of B20 storage tanks in operation at two locations, identified the microorganisms associated with fuel fouling, and measured *in situ* corrosion. The bacterial populations were more diverse than the fungal populations, and largely unique to each location. The bacterial populations included members of the *Acetobacteraceae, Clostridiaceae*, and Proteobacteria. The abundant Eukaryotes at both locations consisted of the same taxa, including a filamentous fungus within the family *Trichocomaceae*, not yet widely recognized as a contaminant of petroleum fuels, and the *Saccharomycetaceae* family of yeasts. Increases in the absolute and relative abundances of the *Trichocomaceae* were correlated with significant, visible fouling and pitting corrosion. This study identified the relationship between fouling of B20 with increased rates of corrosion and the microorganisms responsible, largely at the bottom of the sampled storage tanks. To our knowledge this is the first *in situ* study of this scale incorporating community and corrosion measurements in an active biodiesel storage environment.

## Introduction

The use of renewable fuels is on the rise worldwide (Beiter and Tian, 2016). One renewable fuel in use broadly is biodiesel, which is composed of fatty acid methyl esters (FAME) derived from plant and animal lipids. Biodiesel is commonly used as a replacement or additive to ultra low sulfur diesel (ULSD) up to 5% v/v to restore lubricity lost due to the removal of sulfur compounds (Steidley and Knothe, 2005; Moser, 2009; Gill et al., 2012). Blends containing up to 20% v/v biodiesel (i.e., B20) can be used without changes to existing engines or infrastructure (U.S. Air Force Energy Strategic Plan [USAF], 2010). Combustion of these blends produces reduced nitric oxide emissions, an ozone precursor of concern, than pure biodiesel fuel (Basha et al., 2009). The consumption of biodiesel has increased worldwide over the past 10 years because of its combustion properties (Chang et al., 1996; Canakci, 2007), performance advantages, and increasingly strict emissions regulations (Blakeley, 2012).

The advantages offered by blending biodiesel with ULSD are potentially offset by the drawback of lower fuel stability (Lisiecki et al., 2014). Biodiesel contains more dissolved oxygen than ULSD, reducing oxidative stability and increasing the risk of fuel biodegradation (Speidel et al., 2000). Biodiesel is also more hygroscopic than ULSD (Fregolente et al., 2012), causing blends like B20 to absorb and retain more water. Water in fuel represents habitat for microorganisms and is essential for microbial metabolism and growth; water entrained within fuel enables microbiological proliferation, resulting in fouling. Liquid water is denser than fuel and will accumulate at the bottom of any storage vessel. Microorganisms at the fuel-water interface are able to live in an aqueous environment with the fuel as an oxidizable substrate and source of dissolved oxygen (Prince et al., 2008). Fouling (e.g., the accumulation of biomass) is greatest at the fuel-water interface and microbial metabolism quickly depletes any oxygen in the water (Prince et al., 2008; Owsianiak et al., 2009; Bucker et al., 2011; Passman, 2013).

Microbial growth and subsequent corrosion are dependent upon fuel chemistry and the amount of water present (Aktas et al., 2010; Lee et al., 2010). Biodiesel and biodiesel blends do not contain free fatty acids by specification, but copious amounts of fatty acids are produced as bi-products of the metabolism of FAME by microorganisms (Aktas et al., 2010; Yassine et al., 2013) that may result in microbiologically influenced corrosion (MIC) of steel tanks or tank components. Polymer tank seals and fittings that are also exposed to organic acids can exhibit decreased ductility and strength. Production of CO_2_ during the microbial metabolism of FAME could also increase the rate of corrosion within a storage tank (Crolet et al., 1999). General production and dissolution of acid and CO_2_ within a fluid can result in uniform corrosion, where the loss of material is distributed across a surface. The accumulation of biofilms results in several possible outcomes including the localized production and concentration of corrosive compounds, the formation of galvanic couples, and the ennoblement of steel surfaces. Any of these would result in localized corrosion that is characterized by deep, penetrating pits on metallic surfaces that represent a great risk to storage tank infrastructure (Lee et al., 2009; Little and Lee, 2014). The link between biodegradation and corrosion has been studied in other petroleum environments such as oil production, drilling (Duncan et al., 2009; Stevenson et al., 2011), or storage of petroleum products in marine environments including biodiesel (Aktas et al., 2010; Lyles et al., 2013) yet no *in situ* study exists to this point in B20 storage and distribution systems.

For over a decade, the United States Air Force (USAF) has stored B20 biodiesel and used it in non-tactical support vehicles as a component of their Strategic Energy Plan (Blakeley, 2012). The adoption of B20 biodiesel by the USAF, however, correlated with an increase in reports of “bad fuel,” or fouling due to visible particulates in the fuel and clogged filters at dispensers. We hypothesized that the reported fouling was microbiological in origin, rather than from the abiotic production of waxes in fuel that was suspected by fuel operators and reported previously (Tang et al., 2008; Botella et al., 2014). Both fuel fouling and MIC have the potential to impact the operation of B20 biodiesel storage and distribution systems. Pure cultures and enrichments of microorganisms have shown the ability to degrade biodiesel blends (including B20) and induce corrosion under laboratory conditions (Prince et al., 2008; Bucker et al., 2011; Wang et al., 2011) but no studies link the microbial community and its proliferation with corrosion in active storage tanks over time. To this end, we conducted a rare longitudinal study, *in situ*, of microbial community dynamics linked to the corrosion of steel in B20 biodiesel underground storage tanks (UST). Two USAF locations housing B20 biodiesel tanks with recurrent fuel quality issues were selected for this study. We hypothesized that the reported issues were the result of microbial contamination and predicted that corrosion would be greatest in tanks with the most biomass. We subsequently conducted a 1-year survey of the microbial communities present at both storage locations to link microbiological fouling, fuel degradation, and MIC *in situ* within each storage tank.

## Materials and Methods

### Description of Sampling Sites

The yearlong longitudinal study described here was conducted at two USAF bases, which were located in the SE and SW United States. Each base had three storage tanks containing B20 biodiesel (total of 6 tanks) that were under normal operation throughout the study. The only exception was tank SE 3, which was removed from the study after 9 months due to severe microbiological contamination that required mitigation. These tanks were not on a defined cleaning schedule. Instead, they were taken offline and cleaned as fouling or contamination was detected. All tanks at SW and two tanks at SE (SE 3 and 4) were cleaned just prior to the onset of the study and insertion of corrosion or “witness” coupons. The sampled tanks had an atmospheric (i.e., oxic) headspace and no biocides were used in the fuels stored at each location. The three tanks at SW were constructed of uncoated carbon steel and installed in the early 1950s. At the SE site, two of the tanks (SE 3 and SE 4) were fiberglass and located at the same fueling station. The third tank (SE E) was made of carbon steel, lined on the outside with fiberglass, and located at another fueling station. Each of the six tanks had a large maintenance hatch (i.e., “manway”) that was used as an access point for access and fuel sampling (Supplementary Figure S1). A visual overview of the sampling protocol, including references to the relevant methods sections is included for clarity as Supplementary Figure S3.

### *In situ* Incubation and Sampling of Corrosion Coupons

A PVC rack was used to suspend materials in the fuel phase (60 cm from bottom of tank, top of rack) and in contact with any water phase if it existed (1 cm from bottom of tank, bottom of rack) (Supplementary Figure S2). Four types of materials typical of fuel systems were exposed to these *in situ* conditions: pre-weighed uncoated and epoxy-coated steel (ACT Test Panel LLC., Hillsdale, MI, United States) test specimens commonly known as “witness coupons,” and O-Rings made of V747-75 fluorocarbon or N602-70 nitrile, polymers commonly used in fuel systems (Personal communication, Air Force Petroleum Office). Epoxy coated coupons were prepared by the AFRL Coatings Technology Integration Office (Wright-Patterson AFB, OH, United States) using three coats- (1) A base of MIL-P-24441/20 epoxy/polyamide, formula 150 type III; (2) An intermediate coat of MIL-P-244413/30 epoxy/polyamide, formula 151 type IV; and (3) A topcoat of MIL-P-24441/31 epoxy/polyamide, formula 152 type IV. The metal test specimens (uncoated, *n* = 396, and epoxy-coated steel, *n* = 283) measured 2.54 cm × 7.62 cm were divided evenly between the top and bottom of each rack and removed at ≈3-month intervals at SE, or ≈6-month intervals at SW. As a control, sterile, uncoated witness coupons and sterile O-rings were incubated separately in 0.22 μm filter-sterilized B20 taken from fuel samples received at each site prior to exposure to storage tanks (*n* = 9 for each location).

Replicate witness coupons (*n* ≥ 5) were removed from each underground storage tank (UST) at specified time intervals and photographed on-site. Sampled coupons were subjected to several types of analyses: characterization of biofilm microbial communities by DNA sequencing, microscopy, and quantitation of uniform and pitting corrosion by mass loss and microscopy, respectively. A schematic representation of the sampling workflow is shown in Supplementary Figure S3. Biofilms were sampled for molecular community analyses from triplicate (*n* = 3) witness coupons immediately following their removal from the tank as detailed below. Any biofilm present on the remaining two (*n* = 2) coupons was left intact. Otherwise, all coupons were stored in a desiccating environment to prevent additional corrosion and shipped overnight prior to further analyses.

### Sampling, DNA Extraction, Small Subunit rRNA Gene Library Preparation, and Sequencing of Microbial Biomass

Samples of both biofilms and fuel were taken at each timepoint for molecular analyses, as summarized in Supplementary Figure S3. For molecular analyses of biofilm, the surface of each coupon was immediately sampled after removal from the tank using nylon flocked swabs (Therapak Corp, Los Angeles, CA, United States). Each swab was placed into a 2.0 mL ZR BashingBead^TM^ Lysis Tube (Zymo Research Corp., Irvine, CA, United States) containing 0.7 mL (dry volume) of 0.5 mm ZR BashingBead^TM^ lysis matrix (Zymo Research Corp.) and 750 μL Xpedition^TM^ Lysis/Stabilization Solution (Zymo Research Corp.) and homogenized for 30 s on site using a sample cup attached to a cordless reciprocating saw. Fuel samples were collected and processed within 2–4 h.

Fuel samples were taken at each time point from the bottom of each tank via the access hatch using a 500 mL Bacon bomb fuel sampler (Thermo Fisher Scientific, Hampton, NH, United States). Approximately 1 L of fuel was filtered through a 120 mm 0.22 μm polyether sulfone bottle-top filter. At SE, the filtered fuel was also retained for acid-index determination. The resulting filter was quartered after sampling using a sterile scalpel, and three of these quarters were placed into individual ZR BashingBead^TM^ Lysis tubes containing 0.7 mL (dry volume) of 0.5 mm ZR BashingBead^TM^ lysis matrix (Zymo Research Corp.) and 750 μL Xpedition^TM^ Lysis/Stabilization Solution (Zymo Research Corp.), for a total of three technical replicates per sampling. These samples were homogenized for 30 s on site using a sample cup attached to a cordless reciprocating saw, transported overnight at room temperature and stored at −20°C until needed. Prior to DNA extraction, the samples were homogenized for an additional 30 s using a BioSpec Mini-BeadBeater-8 (Biospec Products Inc., Bartlesville, OK, United States). DNA extractions were performed per manufacturer specifications using the Zymo Xpedition^TM^ Kit (Zymo Research Corp.).

Libraries of bacterial, archaeal, and eukaryotic small subunit (SSU) rRNA gene fragments were amplified from each DNA extraction using a single set of PCR with primers that spanned the SSU rRNA gene V4/V5 hypervariable regions between position 515 and 926 (*E. coli* numbering) as described previously (Parada et al., 2015; Kraus et al., 2018). The primers used were selected to evenly amplify all three domains of life simultaneously (Parada et al., 2015). To mitigate the effects of contaminating DNA (Salter et al., 2014), multiple extraction blanks and negative controls were sequenced from each batch of extractions. All SSU rRNA gene libraries were sequenced using Illumina MiSeq V2 PE250 chemistry at the Oklahoma Medical Research Foundation.

### Analysis of SSU rRNA Gene Sequencing Libraries

Initial quality control, demultiplexing, and OTU clustering at 97 percent sequence similarity was performed as previously described (Stamps et al., 2016), with the modification of using mothur (Schloss et al., 2009) to assign taxonomy against the SILVA database (r128) (Pruesse et al., 2007) formatted for use with mothur. Statistical analyses and figures were generated within R using Phyloseq (McMurdie and Holmes, 2013) and AmpVis (Albertsen et al., 2015). Differences in community composition were estimated using the weighted UniFrac index (Lozupone and Knight, 2005). Datasets were rarefied to 2000 sequences per sample for bacteria/archaea, and 1500 sequences per sample for the eukarya to produce ordinations and perform PERMANOVA analyses. The effect of site, tank, material, and the location of materials within each tank (top or bottom) were tested by a PERMANOVA within the R package Vegan (Dixon, 2003). Sequencing reads are available under the accessions SRR5826605-SRR5826609. The mapping file necessary for demultiplexing in QIIME is available in Supplementary Table S1.

### Estimation of Final Coupon Biomass

The growth of biofilms within the tanks was determined by measuring the biomass attached to the polymer-coated witness coupons. After 12 months of exposure (9 months for SE 3), all pre-weighed polymer coated coupons were removed from each UST at both locations, photographed on site, placed into sterile 50 mL conical tubes, and shipped overnight for further analysis. Upon arrival, coupons were desiccated to remove any water or fuel, weighed, and then cleaned using the ASTM standard G1-03 hydrochloric acid method (G01 Committee, 2012). Briefly, coupons were sonicated for 10 min in soapy water, rinsed in deionized water, dried, and incubated in 3 M hydrochloric acid with 3.5 g/L hexamethylene tetramine solution for 10 min. Acid cleaned coupons were washed in deionized water, acetone, and methanol, dried under a stream of nitrogen, and weighed. Biomass at 12 months was estimated as the difference in the weight of the coupons prior to cleaning (after desiccation) and after cleaning. No obvious degradation of the polymer coating was observed visually before or after removal of the biofilm from sampled coupons.

### Microscopy and Determination of Corrosion on Witness Coupons

Biofilms were visualized on undisturbed uncoated steel witness coupons (*n* = 2 per UST, per timepoint) by optical and electron microscopy prior to coupon cleaning. For determination of general corrosion, biofilm and corrosion products were removed from coupons (*n* = 5 per UST, per timepoint) via ASTM standard G1-03 C.3.5 (G01 Committee, 2012) as detailed in section “Analysis of SSU rRNA Gene Sequencing Libraries.” After cleaning, the mass of each coupon was compared to its pre-exposure mass to determine mass loss. A subset of steel witness coupons (*n* = 2 per time point) was imaged by several complementary approaches: SEM, confocal microscopy, and profilometry. Coupons with intact biofilms that were intended for microscopic analysis were scored with a 2 × 14 grid to enable correlation between locations imaged prior to the removal of biomass and after imaging for pitting corrosion via profilometry (each quadrant roughly 9 × 11 mm). Select coupons were then imaged with the biofilm in place using a VHX 2000E (Keyence Corp., Itasca, IL, United States) digital microscope from 20 to 200× magnification. Areas of interest were subsequently imaged using an FEI Quanta 600 environmental scanning electron microscope (ESEM, Thermo Fisher Scientific Co., Waltham, MA, United States). Three to five areas were imaged with SEM per coupon.

Coupons designated for pitting measurement were imaged after cleaning using a VK-X250 3D Laser Scanning Confocal Microscope (Keyence Corp., Itasca, IL, United States) to measure the depth of each pit and surface roughness. Each coupon was imaged under 10× magnification at 42 identical locations. Maximum pitting rates (recorded as MPY) were calculated for each sample point by the formula $\frac{D\,e\,e\,p\,e\,s\,t\,P\,i\,t\,\left(m\,m\right)\times 365}{E\,x\,p\,o\,s\,u\,r\,e\,T\,i\,m\,e\,\left(D\,a\,y\,s\right)}$ to calculate the rate of corrosion in mm/year and then converted to a value of MPY by multiplying the result by 39.4 (1 mm/y = 39.4 MPY). Pits were defined as a region that was a minimum of 10 μm below the mathematically determined reference plane (surface) and at least 200 μm^2^ in area. A Shapiro-Wilk (Shapiro and Wilk, 1965) test of normalcy (*p* > 0.05) was used to determine normality of the dataset, after which a non-parametric Van der Waerden test (Van Der Waerden, 1953) was used to compare field coupons to sterile controls. Multiple pairwise tests comparing *in situ* incubations to controls were carried out *post hoc* using a Conover test (Gellings and Gudger, 2012) within the package PMCMR, and *p* values were corrected using the false discovery rate (FDR) method (Yekutieli and Benjamini, 2001) within PMCMR. Correlation between the presence of biofilm and corrosion was determined using coupons scored with a diamond knife to facilitate sample orientation, which were imaged by SEM and by confocal microscopy VK-X250.

### Tensile Testing of Polymeric Seal Materials

At each time point, O-ring specimens (*n* = 3) were removed from each tank and the sterile controls, photographed on-site, and the accumulated biofilm was removed prior to testing. The cleaned O-rings were placed into sterile 50 ml conical tubes and shipped overnight for analysis. Tensile testing (strength and elongation) of the O-rings was conducted at the University of Dayton Research Institute in accordance with the test methods and procedures referenced in ASTM D1414 and included analyses of percent elongation, tensile strength, and maximum load.

### Determination of Fuel Acid Index

The acid index of fuel samples taken from the bottom of tanks at the SE location was measured by acid titration using the ASTM standard D974 (D27 Committee, 2008) method at the 3, 7, and 9 months time points. Approximately 20 g of B20 suspended in 100 mL of titration solvent (100:1: 99 Toluene/Water/Isopropyl alcohol) and 0.5 mL of an indicator solution was titrated using a 0.1 N solution of KOH dissolved in isopropyl alcohol (Sigma Aldrich).

## Results

### Microbial Diversity and Taxonomic Composition

Surface samples taken at each time point in each UST from coated coupons, uncoated coupons, and O-rings (representing biofilm communities), and filter quarters (representing fuel communities) were sequenced after sampling. After clustering and quality control, 3.04 million reads remained, which clustered into 759 OTUs across all three domains of life from 306 samples (Supplementary Table S2). Mean library size for Bacteria and Archaea was 6857 reads, and 3470 for the Eukarya (Supplementary Table S2). Due to low sequence count, after rarefaction 59 samples and 9 OTUs were removed from beta diversity analyses within the bacterial/archaeal dataset, while 76 samples and 29 OTUs were removed from the eukaryotic dataset.

**FIGURE 1:**
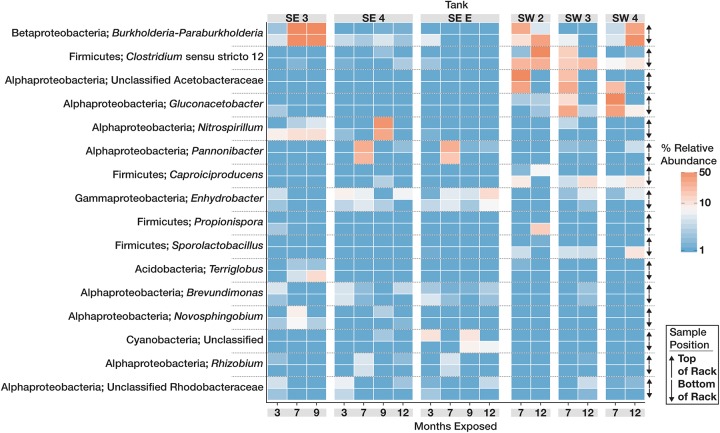
Relative abundance heat map showing the relative abundance of the 20 most abundant bacterial OTUs within the microbial communities found in biofilms within each tank over time. For each taxon shown, the heat map is divided into samples which were suspended from the top and bottom of the PVC rack. Taxonomic identity for each OTU is shown as the most likely genus (if identifiable) and phylum. For clarity, Proteobacterial classes are shown in place of the phylum designation.

**FIGURE 2:**
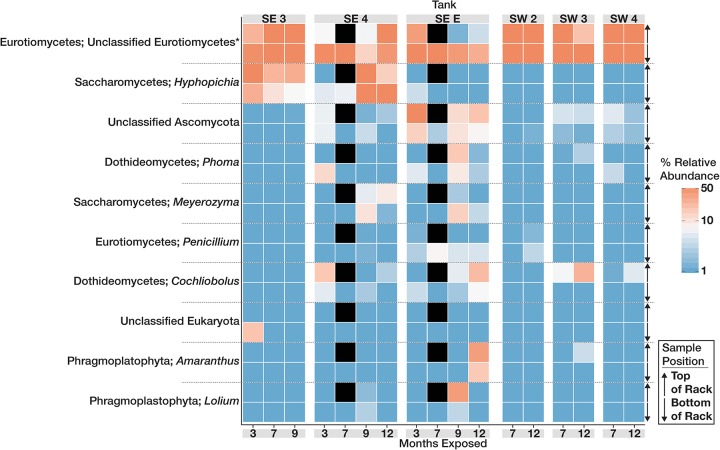
Heat map showing the relative abundance of the 10 most abundant Eukaryotic OTUs within the microbial communities found in biofilms within each tank over time. For each taxon shown, the heat map is divided into samples which were suspended from the top and bottom of the PVC rack. Taxonomic identity for each OTU is shown as the most likely genus (if identifiable) and phylum. Black boxes denote that insufficient numbers of sequence were avalible to analyze for those timepoints.

The community structure and composition of sampled biofilms varied significantly between bases (Bacteria *p* = 0.001; *R*^2^ = 0.12, Eukaryota *p* = 0.001; *R*^2^ = 0.23) and among different tanks at each base (Bacteria *p* = 0.001; *R*^2^ = 0.37, Eukaryota *p* = 0.001; *R*^2^ = 0.34). Members of the Acetobacteraceae (acetic acid bacteria) or the Clostridiaceae group 1 were the most abundant taxa detected in the tanks at SW, whereas OTUs most closely related to the genera *Pannonibacter* (Family Rhodobacteraceae) and *Nitrospirillum* (Family Rhodospirillaceae) were more abundant in SE tanks (Figure 1). An OTU (OTU 1 within the Eukaryotic dataset) that was present and often very abundant at both locations was identified as an unclassified member of the Eurotiomycetes. Additional identification via BLAST suggested that it was a filamentous fungus, most likely a member of the genus *Byssochlamys* (Family Trichocomaceae). The microbial communities at SE also intermittently contained a population of yeast most closely related to the genera *Saccharomyces* and *Wickerhamomyces* (Family Saccharomycetaceae) (Figure 2). A summary of all detected taxa can be found in Supplementary Table S2.

**FIGURE 3:**
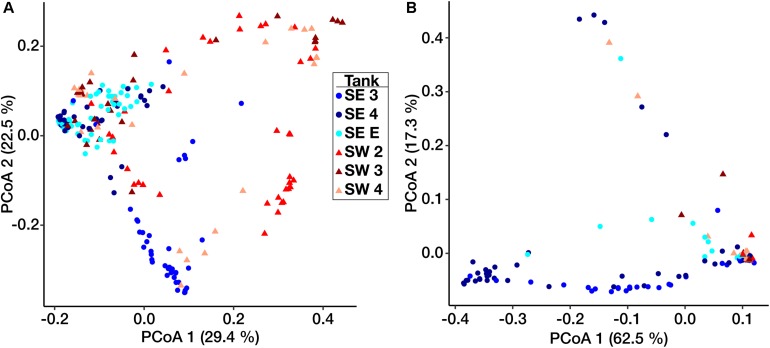
Weighted UniFrac principal coordinate ordination of the Bacterial **(A)** and Eukaryotic **(B)** biofilm communities. Points are separated by location – SE (circles) and SW (triangles). Colors represent individual tanks at each location.

Ordination based on Principal Coordinates Analysis (PCoA) using the weighted UniFrac distance among microbial communities including both fuel and biofilms indicated that these communities differed by site (Figure 3). Eukaryotic samples from SW grouped closely together visually by ordination compared to those from SE, which clustered more closely by tank rather than location (Figure 3B). Small differences were detected between samples of fuel and biofilm at each sampling point among the bacteria (Supplementary Figure S4A), but there was little to no difference among the eukarya (Supplementary Figure S4B).

### Site Observations, Biomass, and Corrosion Measurements

Total dry weight biomass after 12 months of exposure to fouled fuel was greatest at SW, with SW 2 having the greatest fouling of coupon surfaces (Supplementary Figure S5). Overall, coupons at SW were consistently coated in greater amounts of biofilm/material (Supplementary Figure S6) and had greater final biomass than those sampled at SE.

**FIGURE 4:**
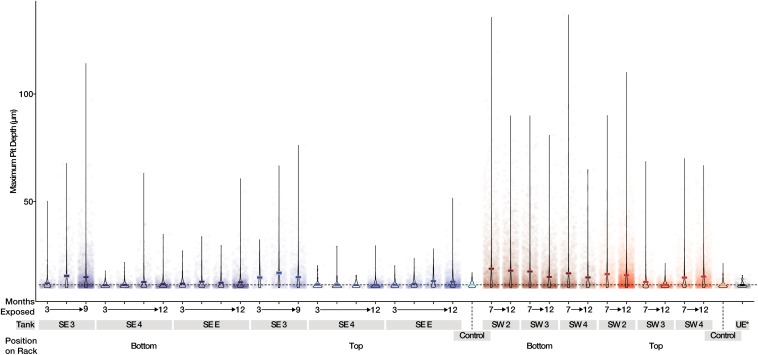
Maximum pit depth measured for uncoated steel witness coupons from SE (Blue) and SW (Red). Mean values are shown as a dark line for each sample. The dashed line represents the mean of witness coupons not exposed to fuel or field conditions.

A total of 242 coupons (including controls) were used in the analysis of mass loss, and 280 coupons (including controls) were used for pitting and roughness analyses between bases. The pitting analyses generated a total of 37,289 measurements from the 280 coupons and surface roughness analyses generated a total of 12,810 measurements from the 280 sampled coupons. Witness coupons from tanks at SW consistently experienced greater amounts of corrosion than those at SE (Supplementary Figure S7). All coupons exposed to SW tank systems had significantly more corrosion than controls exposed to sterile fuel. At SE both SE 3 and SE E had significantly more corrosion than controls while SE 4 coupons were not significantly different from sterile fuel controls (Supplementary Table S3). Coupons exposed to conditions in SW tanks had deeper pits than coupons from the SE location and controls (Figure 4). Over time, the skewedness of pit distribution on exposed coupons was greater than the controls (Figure 4). Surface roughness also increased over time (Supplementary Figure S8) and was the greatest on coupons from SW 2. Tanks at SW, and SE 3 had the highest pitting rate (Table 1). Corrosion rates on the bottom coupons in SW 2 and SW 4 bottom exceeded 8 MPY. Compared to coupons attached to the top of the rack, coupons placed at the bottom of tanks had deeper pits (Figure 4, *p* < 0.001), but surface roughness was not significantly different (*p* = 0.34, Supplementary Figure S8). There was little to no effect on load strength, tensile strength, or elongation among tanks of o-rings exposed to fuel at either the top or the bottom of the rack (Supplementary Figure S9).

**TABLE 1:** Maximum pitting rate in mils per year (MPY) calculated from the deepest pit observed in each tank, at each time point.

**Exposure Time (Months)**	**3**	**7**	**9**	**12**
**Tank**				
SE 3 (Top)	5.04	4.73	4.18	-
SE 3 (Bottom)	7.86	5.04	6.27	-
SE 4 (Top)	3.17	2.04	0.86	1.11
SE 4 (Bottom)	2.82	1.52	3.47	1.32
SE E (Top)	3.17	1.62	1.5	2.3
SE E (Bottom)	4.25	2.31	1.58	1.95
SW 2 (Top)	-	5.59	-	4.03
SW 2 (Bottom)	-	8.4	-	3.29
SW 3 (Top)	-	4.25	-	0.78
SW 3 (Bottom)	-	5.57	-	2.95
SW 4 (Top)	-	4.34	-	2.44
SW 4 (Bottom)	-	8.46	-	2.38
SE Control	-	-	-	0.64
SW Control	-	-	-	0.78

**FIGURE 5:**
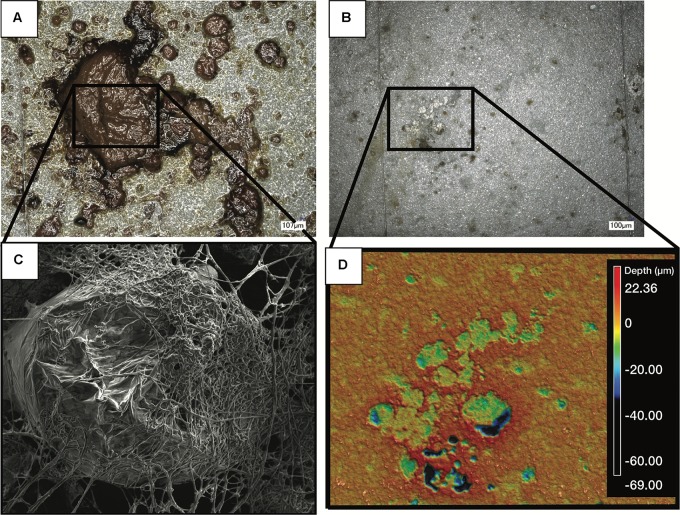
Images of a representative biofilm **(A)** on a witness coupon. After cleaning of the same biofilm, pitting corrosion was evident underneath the biofilm **(B)**. SEM imaging of the coupon before cleaning revealed a large mass of fungal hyphae **(C)**, while profilometry of the cleaned coupon showed clear pitting corrosion underneath the fungal biofilm **(D)**.

The acidity of fuel in SE 3, measured as acid number, increased over time from 0.17 to 1.51 mg KOH/g B20. The elevated acidity corresponded elevated corrosion rates and maximum pit depths in SE tank 3 relative to other tanks at SE. By 9 months, fuel in SE tank 3 exceeded the ASTM standard limit of 0.3 mg KOH/g B20 for fuel acidity (Table 2). Witness coupons imaged by SEM from both bases were covered by morphologically similar biofilms (Figure 5A), which were primarily comprised of fungal hyphae (Figure 5C). Pits on the coupon surfaces were correlated with areas that contained biofilm prior to cleaning (Figures 5B,D).

**TABLE 2:** Acid index of select fuels from SE at 3, 7, and 9 months^*a*^.

	**3 Months**	**7 Months**	**9 Months**
SE 3	0.17	0.30	**1.51**
SE 4	0.08	0.16	0.28
SE E	0.22	0.09	0.24
Receipt^*b*^			0.06

*^*a*^Values are expressed in mg KOH/g B20. Bold values indicate fuel exceeds ASTM standard for acidity. ^*b*^Receipt was fuel taken directly from a delivery truck before exposure to a storage tank.*

## Discussion

The advantages of blending or replacing ULSD with biodiesel include increased fuel performance and significant reductions in carbon emissions (Gill et al., 2012). Enhanced fuel standards have resulted in a worldwide increase in biodiesel consumption from 2.2 million liters per day (lpd) in 2000 to 84.3 million lpd in 2014 (Beiter and Tian, 2016). The USAF started using B20 biodiesel in non-emergency, non-tactical ground vehicles and consumed 12.1 million liters of biodiesel per year in 2010 (U.S. Air Force Energy Strategic Plan [USAF], 2010). A potentially significant disadvantage to their adoption of B20 biodiesel was an increase in reports of fuel biocontamination. The main objectives of this study were to characterize the microbial communities responsible for fouling of B20 biodiesel and determine whether their proliferation and biofilms are associated with increased rates of corrosion of in-service B20 storage tanks.

Microorganisms can more readily metabolize the FAME in biodiesel blends, leading to fouling in fuel storage systems (Prince et al., 2008). Fuel fouling and degradation are not the only concerns, as increased rates of corrosion have been observed when microorganisms proliferate in biodiesel under laboratory conditions (Aktas et al., 2010; Lee et al., 2010). A recent EPA study reported moderate or severe corrosion in 83% of inspected storage tanks (US EPA, 2016), suggesting an additional impact on fuel infrastructure could be linked to the degradation of biodiesel. Recent work has identified the metabolism of microorganisms in biodiesel blends that results in biodegradation (Bücker et al., 2018) but microbial communities in non-marine environments have not yet been directly linked to increased corrosion rates *in situ*. In this work, we conducted the first longitudinal study co-locating the microorganisms known to be responsible for fuel fouling, acid production, and microbially influenced corrosion on “witness” coupons installed near the bottom of tanks *in situ*. Our study established a link between fuel fouling and increased biofilm biomass comprised of specific fungal and bacterial taxa in operational B20 biodiesel tanks. We also linked biomass within B20 storage tanks to pitting corrosion, co-located with the microbial biofilms. The direct linkage between fuel fouling and corrosion is of considerable concern to tank operations.

The most damaging MIC is associated with multispecies biofilms, where interactions between different microorganisms can create a cascade of biochemical reactions between the oxic and anoxic regions (Videla and Characklis, 1992; Little and Lee, 2014). Microbial biofilms and their associated metabolic activities can exacerbate corrosion via several mechanisms. Under anoxic conditions and in the presence of oxidized sulfur compounds, sulfur reducing microorganisms can cause corrosion of iron by either removing electrons from a hydrogen film on the metal surface (EMIC) or directly from the Fe^0^ itself (CMIC) (Enning et al., 2012; Venzlaff et al., 2012; Enning and Garrelfs, 2014). Very little sulfur would be expected in B20 composed of 80% ultralow sulfur diesel and no sulfate-reducing taxa were detected in our molecular analyses, supporting the conclusion that any form of MIC related to anoxic sulfur metabolism was not occurring in these tanks. Other microorganisms known to participate in CMIC, such as iron-oxidizing bacteria (e.g., *Gallionella* sp., *Leptothrix* sp., and *Mariprofundus* sp.) (McBeth et al., 2011; Lee et al., 2013; Liu et al., 2017) and methanogenic archaea (Enning et al., 2012) were not detected in biofilm or fuel samples.

Biofilms associated with the greatest amount of MIC in our study were composed largely of the filamentous fungus *Byssochlamys* sp. and acid-producing bacteria (e.g., *Gluconacetobacter* sp.). The *Byssochlamys* sp. mycelial hyphae and the biofilm itself most certainly can facilitate the partitioning of areas containing dissolved oxygen and anoxic sites, thus creating an electrochemical oxygen concentration corrosion cell (Videla and Herrera, 2005; Little et al., 2008). Areas of anoxia become anodic sites, where aggressive, localized pitting can occur. Acid production by bacteria and fungi within the biofilm cane also generate localized areas of low pH, further exacerbating the solubilization of metal from the surface (Little and Ray, 2001; Juzeliünas et al., 2007).

We found that the microbial communities forming observable growth in the B20 tanks contained abundant taxa known to oxidize the fatty acids or fatty acid methyl esters present in B20 biodiesel. Limitations for growth of bacteria and fungi in B20 is likely controlled by availability of nitrogen, phosphorus, an electron acceptor, or the presence of water (i.e., physical space at the fuel-water interface). Bacterial taxa potentially capable of nitrogen fixation (*Nitrospirillum* and *Burkholderia*) were present and abundant when biomass was high. Both *Nitrospirillum* and *Burkholderia* have the capacity for nitrogen fixation and thus could be providing the greater microbial community with a source of fixed nitrogen for growth (Estrada-De Los Santos et al., 2001; Chung et al., 2015), living in coexistence with the microorganisms responsible for fuel acidification via the production of organic acids that result in corrosion of metallic surfaces within the tanks.

Aerobic metabolism at the fuel:water interphase would lead to the depletion of dissolved oxygen in water at the bottom of storage tanks. In the absence of other electron acceptors, fermentation would be the main type of microbial metabolism in the water phase, leading to the production of copious organic acids. For example, members of the Firmicutes genus *Caproiciproducens* were abundant in SW tanks and can produce numerous organic acids including caproic, butyric, and acetic acid under anaerobic fermentative growth (Kim et al., 2015). Anaerobic, fermentative microorganisms like the Clostridiaceae (including *Caproiciproducens*), were also abundant at SW in all three tanks.

Aerobic microorganisms, members of the Alphaproteoba cteria that are closely related to *Gluconacetobacter* and unclassified Acetobacteraceae, were abundant in samples from SW tanks. These microorganisms are capable of producing large quantities of acetic acid and producing viscous biofilms of cellulose, which could enhance their ability to maintain a physical presence at the fuel-water interface and exacerbate corrosion and fouling (Castro et al., 2012; Tazato et al., 2012). The presence of aerobic, facultatively anaerobic, and strictly anaerobic bacteria in the tanks is indicative of the sharp gradient in oxygenation. Most likely, the bulk fuel samples taken at the bottom of storage tanks mixed these microorganisms that were otherwise segregated by fine scale gradients of oxygenated fuel and anoxic water.

In contrast to the bacteria, the fungal community was less diverse and more homogenous across all locations. Fungi representing a single, highly abundant OTU closely related to the genus *Byssochlamys* was found in all tanks exhibiting fouling. Members of the genus *Byssochlamys* have been linked to the contamination of acidic fruit juices and are capable of propagating across a broad range of pH and temperatures under oxic conditions (Tournas, 2008). A member of the genus *Byssochlamys* was recently isolated from biodiesel in Hawaii, and characterized for its ability to degrade ULSD and biodiesel (Ye et al., 2017). *Byssochlamys* spores can withstand boiling (high temperatures are a part of the biodiesel production process), low concentrations of oxygen that may exist at the bottom of a fuel tank, and pH values as low as 3.5 as microbial community members begin to acidify water at the bottom of a fuel tank (Tournas, 2008). Additionally, members of the anamorph of *Byssochlamys*, *Paecilomyces*, are known to ferment under anaerobic conditions, producing acetate and ethanol (Mountfort and Rhodes, 1991). Unclassified Eurotiomycetes (identified as *Byssochlamys)* was numerically dominant in all tanks at SW over time with much lower observable diversity than the Bacteria. The abundance of these fungi likely contributes to the increased acidity observed in tanks at SE as well as to the acidification and fouling of fuels at SW. Filamentous fungi such as *Byssochlamys* may also provide a surface for bacteria to exploit, encouraging biofilm production in fuel (Guennoc et al., 2017). SEM analysis of fouled coupons showed co-location of rod-shaped bacteria with the fungal filaments (data not shown). Ours is the first study to conclusively show the biocontamination of B20 biodiesel *in situ* dominated by *Byssochlamys-*like OTUs, and to establish the relationship between their abundance and *in situ* corrosion.

Major differences in community membership were detected between locations and among individual tanks. However, there was no obvious correlation between community membership and environmental (local temperature or rainfall) or operational variables. As a result, we do not have definitive evidence as to why specific microorganisms were associated with a single tank or location. Tank material almost certainly was a factor – corroding steel tanks represent a more reducing environment than the relatively more inert fiberglass tanks, encouraging the growth of anaerobic microorganisms such as *Caproiciproducens* as observed in SW. The tank SW 2 had the greatest amount of corrosion, although another steel tank, SE E, produced much lower amounts of corrosion suggesting that something more than tank material may play a role in the corrosion rate and microbial communities observed.

The data presented in this study highlights the spatial heterogeneity of microbial communities within B20 storage tanks. Most of the detected taxa were found in similar relative abundances both in the top and bottom of each sampled coupon rack, suggesting there is little difference in the microbial communities that occur within these areas of each tank. The greatest differences were observed in SW 2, where differences in the relative abundances of the *Clostridium sensu strictu* 12 and unclassified Acetobacteraceae were noted both over time, and in position within the tank. This could be due to a decrease in the amount of dissolved oxygen both in the fuel and water bottom; OTUs related to *Clostridium* (strict anaerobic microorganisms) increased over time in the upper position while the Unclassified Acetobacterace decreased. Because each storage tank was in operation while it was being monitored, it is also possible that there was a mixing effect on the community as fuel and water levels changed over time, making it difficult to discern differences in community structure between locations in each tank. Shared among all of the sampled tanks, was the presence of a mixed microbial community of both fungi and bacteria that appeared capable of fermentation and the production of organic acids that would increase localized corrosion on metallic surfaces.

The corrosion measured in this study likely resulted from the presence of microbial biofilms causing localized pitting corrosion. The localized production of acids within biofilms created an environment that was favorable for the induction of pitting corrosion similar to that observed within our study (Lee et al., 2010). Our *in situ* study showed greater corrosion near the bottom of each tank where the presence of water and a fuel-water interface was more likely. The interaction of fuel and water allows for enhanced microbial growth, and in turn, greater corrosion than water limited spaces within a fuel tank. Indeed, the greatest corrosion rates observed were in tanks at SW (>8 MPY), where fungal fouling was the most prolific. Rates of corrosion appeared to decline from 7 to 12 months, suggesting passivation may have taken place. Alternatively, the apparent shallowing of pits could be explained by an increase in uniform corrosion, that was observed in SW tanks, which could make pits appear shallower. The exact mechanism of corrosion remains to be deciphered – while steel corrosion is commonly associated with sulfate reduction and the production of sulfide (Lee et al., 2009), no sulfate reducing bacteria were identified within any of the community surveys. The greatest levels of corrosion observed in the SW tanks correlated with the presence of anaerobic *Clostridia* and high relative abundances of OTUs most closely related to *Byssochlamys*, suggesting that corrosion in these tanks was the result of the production of organic acids during the metabolism (either fermentative or aerobic) of FAMEs present within B20. The presence of organic acids produced as a consequence of fuel degradation may also aide in further growth of organisms that can directly incorporate these metabolites into their own cell wall, as was recently observed in an alkane degrading culture (Konieczna et al., 2018). Although we observed biomass including both bacterial and fungi on polymer coated coupons and polymeric O-rings that could degrade or otherwise damage materials (Cregut et al., 2013; Hung et al., 2016), polymer degradation was not detected. It is possible that the polymers tested in this study were resistant to degradation over the period of the study and that a much longer exposure time was required. Our data supports the idea that tank contamination contributes to an increased risk of corrosion but not, apparently, to polymeric degradation.

We can only speculate how the fuel storage tanks became contaminated. All of the tanks sampled, with the exception of SE E, were cleaned just prior to the beginning of *in situ* measurements. Within 6 months, however, a dense fungal mat was visible on coupons removed from many of the tanks. Spores of *Byssochlamys* may enter through the vents from wind-blown dust or enter as a part of the fuel production process in otherwise “clean” fuel. Nevertheless, biofilms were repeatedly found on the *in situ* coupons and sampling racks near the bottom of each tank in a relatively rapid timeframe. It is highly likely that after a tank is colonized, the established community of the tank is the likely source of recurrent contamination, instead of microorganisms immigrating from air vents or water ingress.

## Conclusion

The comprehensive, *in situ* investigation presented here compliments several other laboratory-based studies (Leung et al., 2006; Bucker et al., 2011; Ching et al., 2016). Together, they illustrate the susceptibility of fuels containing biodiesel to microbial proliferation (fouling), fuel biodegradation, and MIC of associated infrastructure. Here though, we were able to *directly* link the presence and prevalence of biofilms to pitting corrosion in actively operating B20 storage tanks. We found that a mixed microbial community of filamentous fungi and acid-producing bacteria were able to proliferate in B20 biodiesel storage tanks, cause fouling, degrade fuel by metabolizing the FAME, produce organic acids, and accelerate steel corrosion under biofilms attached to metal surfaces. The same fungal species was responsible for “blooms” of biomass, providing a target for future mitigation strategies. Additional research is underway to characterize this most abundant fungal member of fuels and biofilms observed at both locations (Stamps et al., 2018).

Despite the trade-offs in fuel stability due to susceptibility to microbial attack, biodiesel continues to be the most common and economical solution to reduce the environmental impact of petroleum diesel combustion. As such, the use of biodiesel will likely continue to increase worldwide for the foreseeable future. Operator vigilance in fuel quality and storage conditions is required including early mitigation through water removal, cleaning, and even biocide treatment. Each of these methods would likely be the most effective means of controlling fouling, degradation, and corrosion through the prevention of microbial biofilm establishment. While B20 presents new storage challenges to operators, risk assessments informed by this study will aid each operator in formulating the appropriate response if contamination is detected.

## Data Availability Statement

The raw sequence data generated for this study can be found in the Sequence Read Archive under the accession numbers SRR5826605–SRR5826609.

## Author Contributions

BWS carried out sampling, experimentation, and wrote the manuscript. CB, CD, JF, and HN carried out sampling, experimentation, and edited the manuscript. PL, KE, and AN carried out experiments and edited the manuscript. WC-G and BSS carried out sampling, edited the manuscript, and conceived of the experiments. All authors have given approval to the final version of the manuscript.

## Conflict of Interest

CD and PL were employed by UES, Inc. KE was employed by Azimuth Corporation.

The remaining authors declare that the research was conducted in the absence of any commercial or financial relationships that could be construed as a potential conflict of interest.

## Supplementary Material

FIGURE S1 Schematic representation of a B20 storage tank. Samples were taken from a “manway” access point (1), and witness coupons were suspended near the bottom of the tank (2), to attempt to expose materials to fuel, as well as to any potential water bottom (3). Other potential ingress points to the tank include the fuel inlet (4) and sampling port (5).

FIGURE S2 Image of a representative coupon sampling rig placed with each tank, prior to exposure.

FIGURE S3 Overview of the workflow for environmental sampling of all sample types, including materials testing of O-rings **(left)**, uncoated carbon steel **(right)**, and all samples destined for DNA extraction **(center)**.

FIGURE S4 Comparison of the Bacterial **(A)** and Eukaryotic **(B)** microbial communities of both fuels and biofilm samples taken at each time point, for each tank.

FIGURE S5 Biomass obtained from coated witness coupons from both SE and SW after one year of exposure within each tank.

FIGURE S6 Images of coupons after removal from tanks at 3, 7, and 9 months (SE), or 7 months (SW).

FIGURE S7 Mass loss obtained over time for each tank at SE (Blue) and SW (Red). Mean values are shown as a dark line for each sample. The dashed line represents the mean of witness coupons not exposed to fuel or field conditions.

FIGURE S8 Roughness (S_*a*_) values of uncoated steel witness coupons from SE (Blue) and SW (Red).

FIGURE S9 O-ring measurements of load **(A)**, tensile strength **(B)**, and elongation **(C)** after exposure to fuels at SE (Blue) and SW (Red). Controls exposed to SE fuel are shown in gray, and unexposed controls are represented in black. Units for load and tensile strength given in lbs/in^2^, and elongation as percent from original length.

TABLE S1 Mapping file of all samples including barcode and primer sequences. Run accessions are given within the table.

TABLE S2 Summary statistics of Bacterial/Archaeal and Eukaryotic small subunit ribosomal RNA gene sequencing libraries **(a)**. Sequence counts and taxonomy strings for both the bacteria/archaea **(b)** and the eukaryotes **(c)** are also given.

TABLE S3 Pairwise significance test results of corrosion both between tanks **(a)** and between tanks subset into individual time points **(b)**.

## Abbreviations

AFB: Air Force Base
AFRL: Air Force Research Laboratory
ASTM: American Society for Testing and Materials
B20: 80:20 blend of ULSD and biodiesel
CMIC: chemical microbiologically induced corrosion
EMIC: electrical microbiologically induced corrosion
FAME: fatty acid methyl ester
MIC: microbiologically influenced corrosion
MPY: mils per year
OTU: operational taxonomic unit
PVC: poly-vinyl chloride
SE: Southeast United States
SEM: scanning electron microscopy
SSU rRNA: small subunit ribosomal RNA
SW: Southwest United States
ULSD: ultra low sulfur diesel
USAF: United States Air Force
UST: underground storage tank.
