# Supplementary figures and images for: In situ Linkage of Fungal and Bacterial Proliferation to Microbiologically Influenced Corrosion in B20 Biodiesel Storage Tanks
Source: Front Microbiol. 2020 Feb 25;11:167. doi: 10.3389/fmicb.2020.00167 (PMC7055474; doi:10.3389/fmicb.2020.00167)

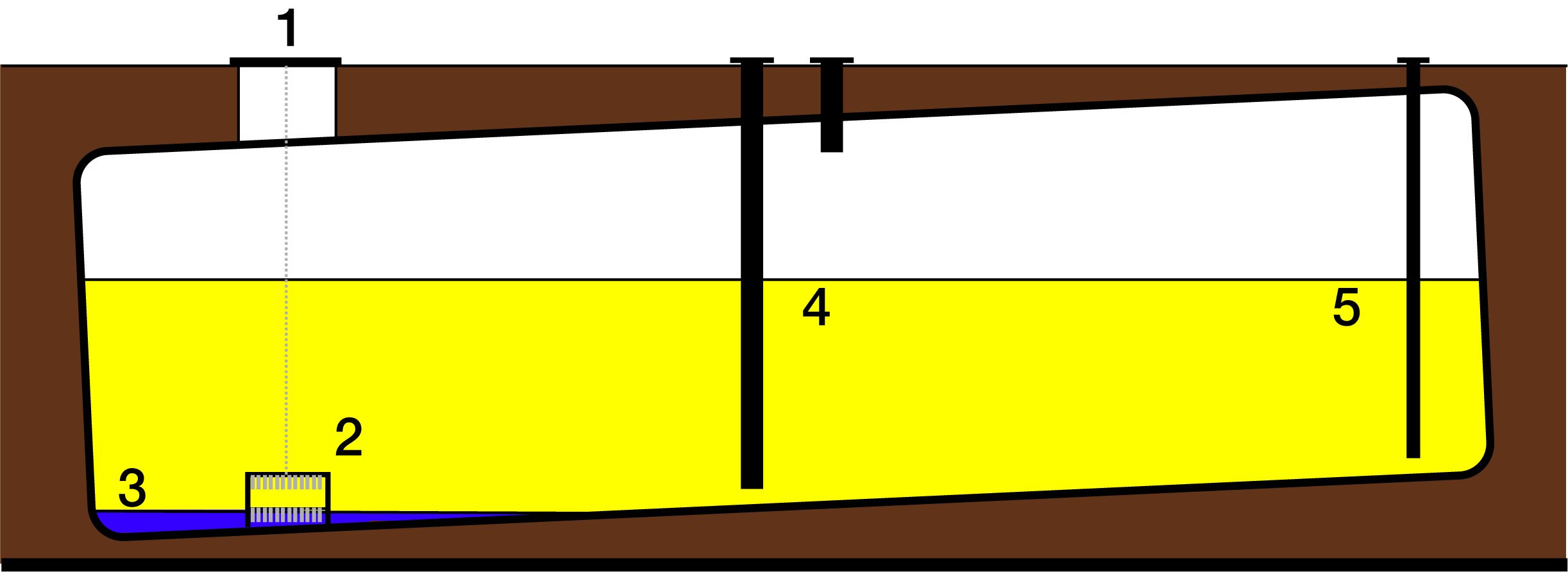

Supplement: FIGURE S1 — Schematic representation of a B20 storage tank. Samples were taken from a “manway” access point (1), and witness coupons were suspended near the bottom of the tank (2), to attempt to expose materials to fuel, as well as to any potential water bottom (3). Other potential ingress points to the tank include the fuel inlet (4) and sampling port (5). [file Image_1.TIF]

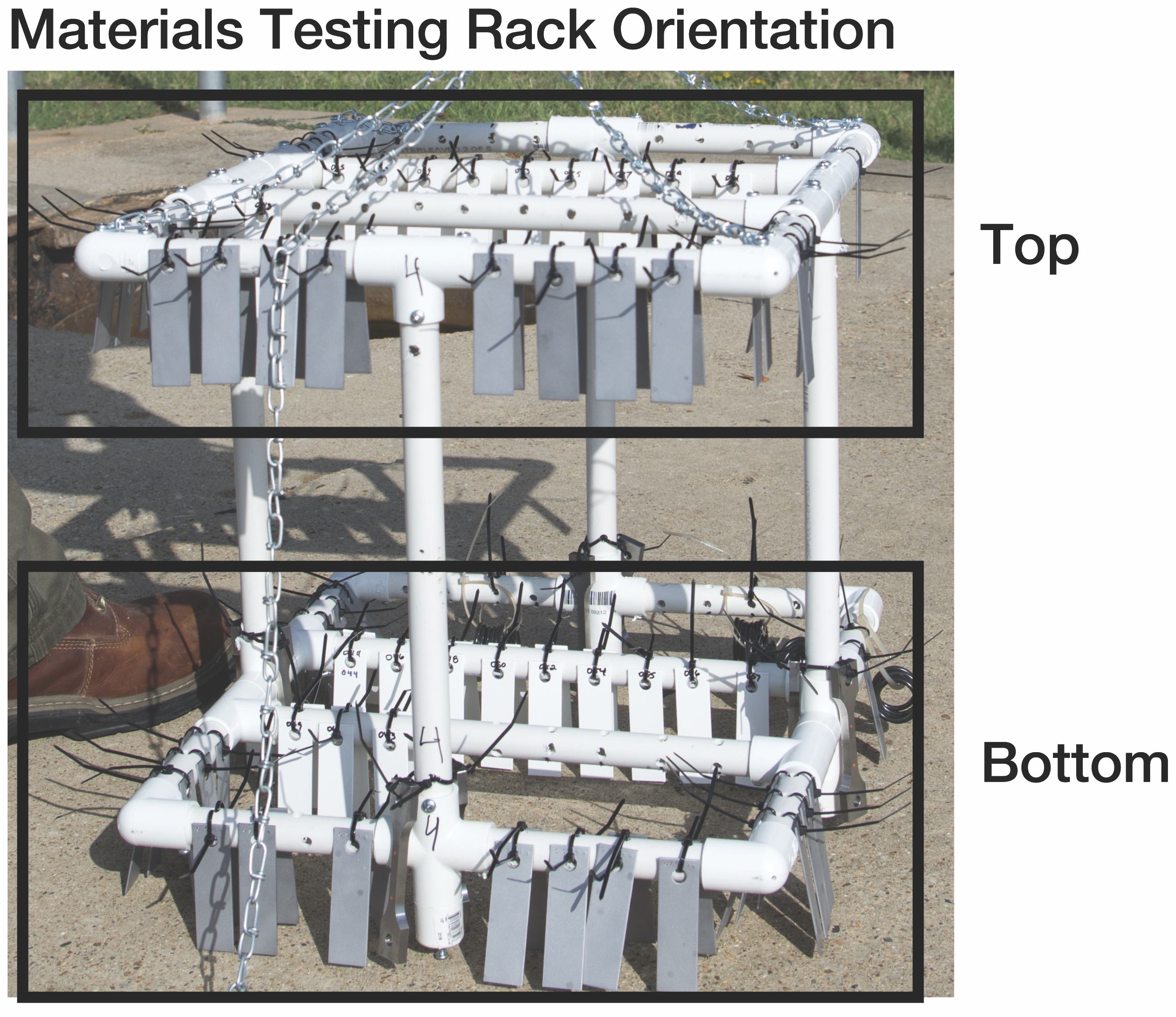

Supplement: FIGURE S2 — Image of a representative coupon sampling rig placed with each tank, prior to exposure. [file Image_2.TIFF]

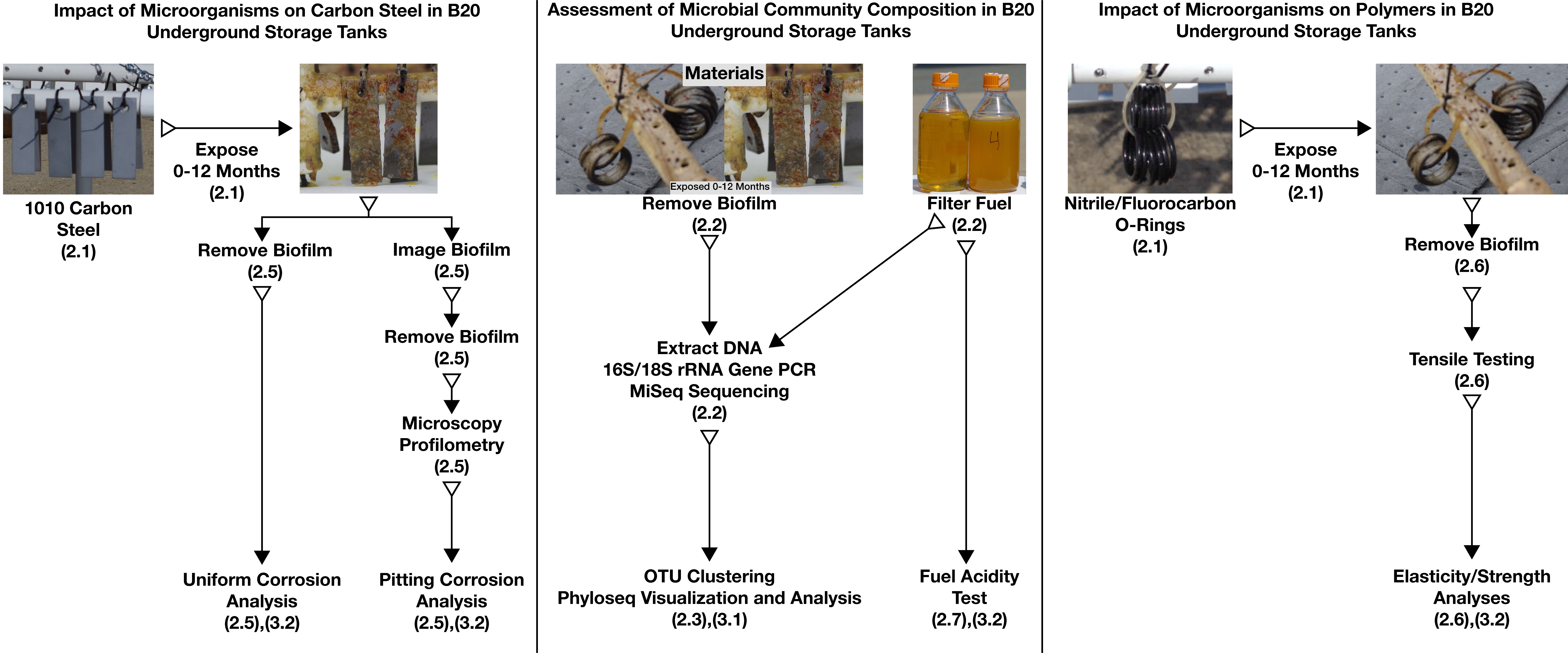

Supplement: FIGURE S3 — Overview of the workflow for environmental sampling of all sample types, including materials testing of O-rings (left), uncoated carbon steel (right), and all samples destined for DNA extraction (center). [file Image_3.TIF]

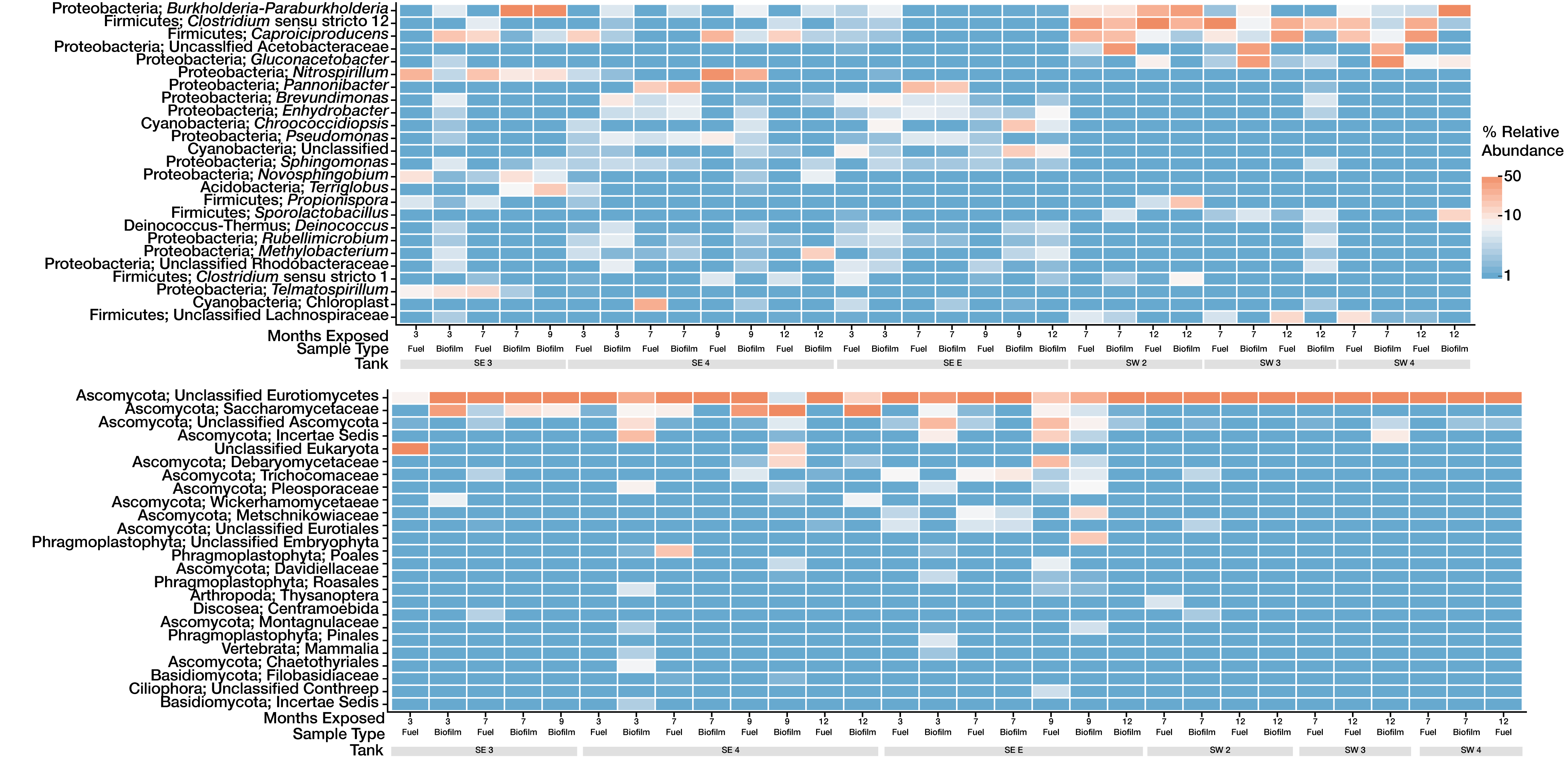

Supplement: FIGURE S4 — Comparison of the Bacterial (A) and Eukaryotic (B) microbial communities of both fuels and biofilm samples taken at each time point, for each tank. [file Image_4.TIF]

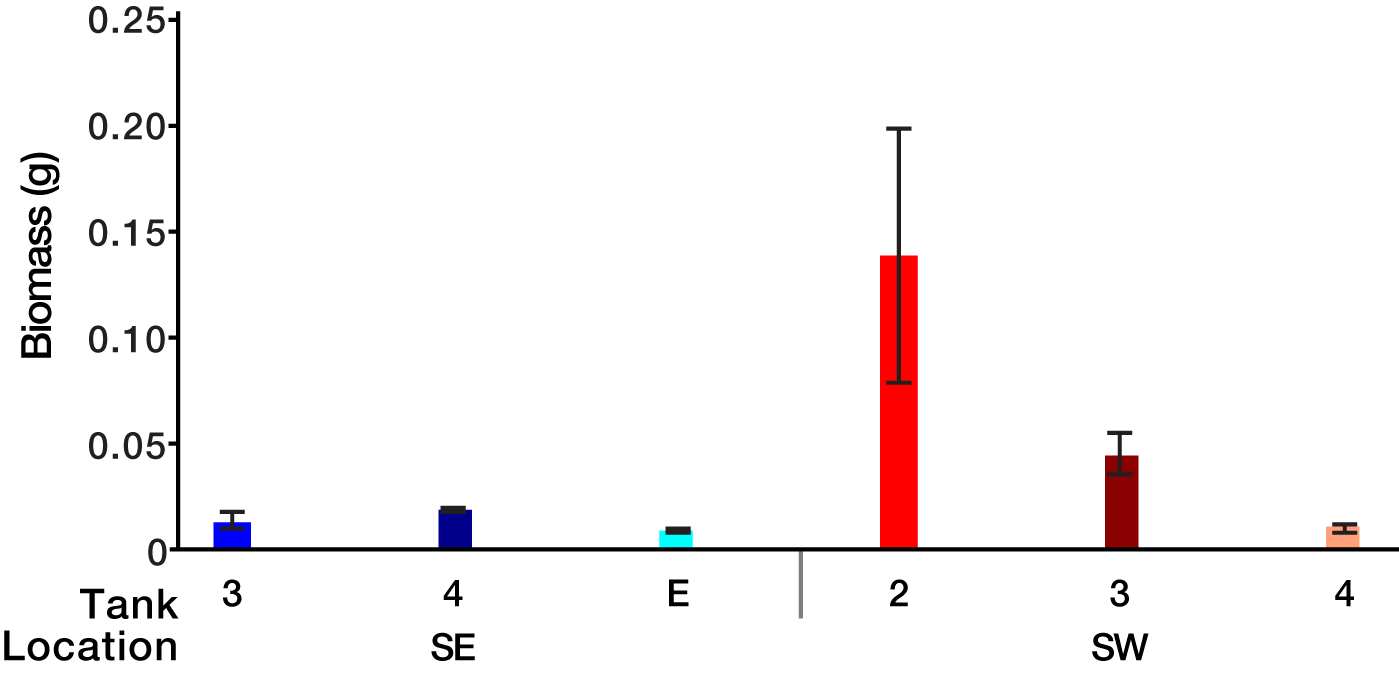

Supplement: FIGURE S5 — Biomass obtained from coated witness coupons from both SE and SW after one year of exposure within each tank. [file Image_5.TIF]

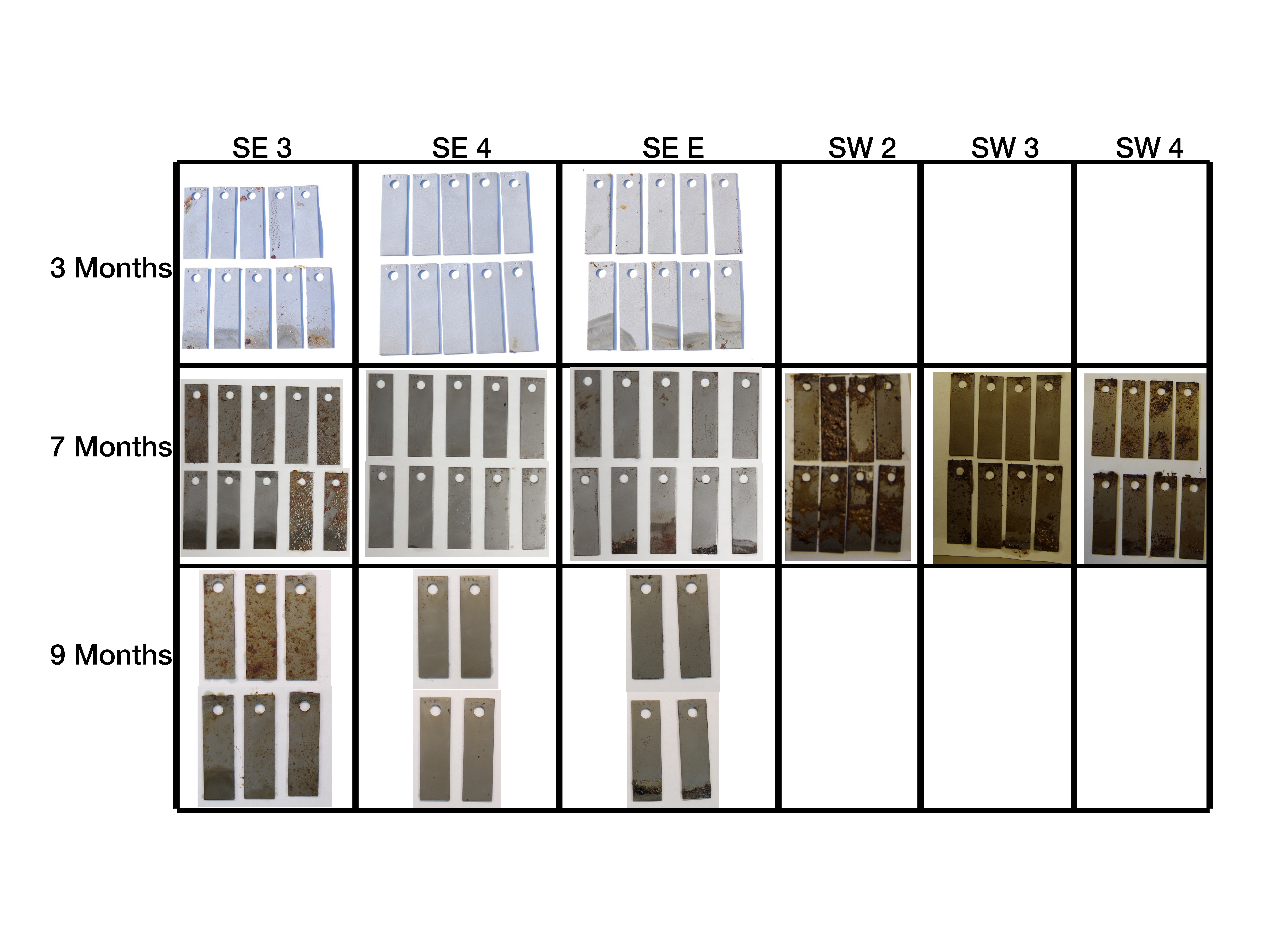

Supplement: FIGURE S6 — Images of coupons after removal from tanks at 3, 7, and 9 months (SE), or 7 months (SW). [file Image_6.TIF]

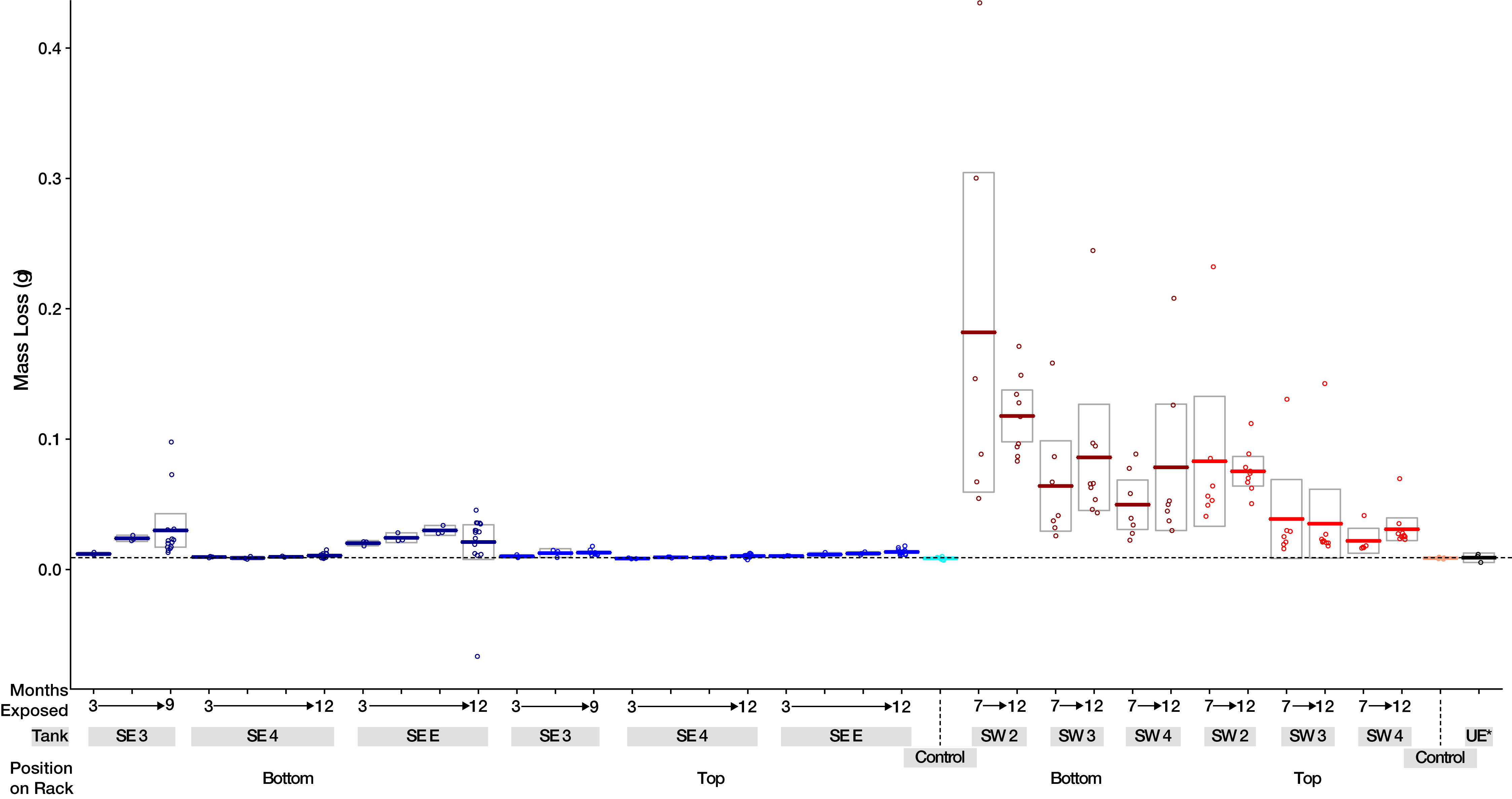

Supplement: FIGURE S7 — Mass loss obtained over time for each tank at SE (Blue) and SW (Red). Mean values are shown as a dark line for each sample. The dashed line represents the mean of witness coupons not exposed to fuel or field conditions. [file Image_7.TIF]

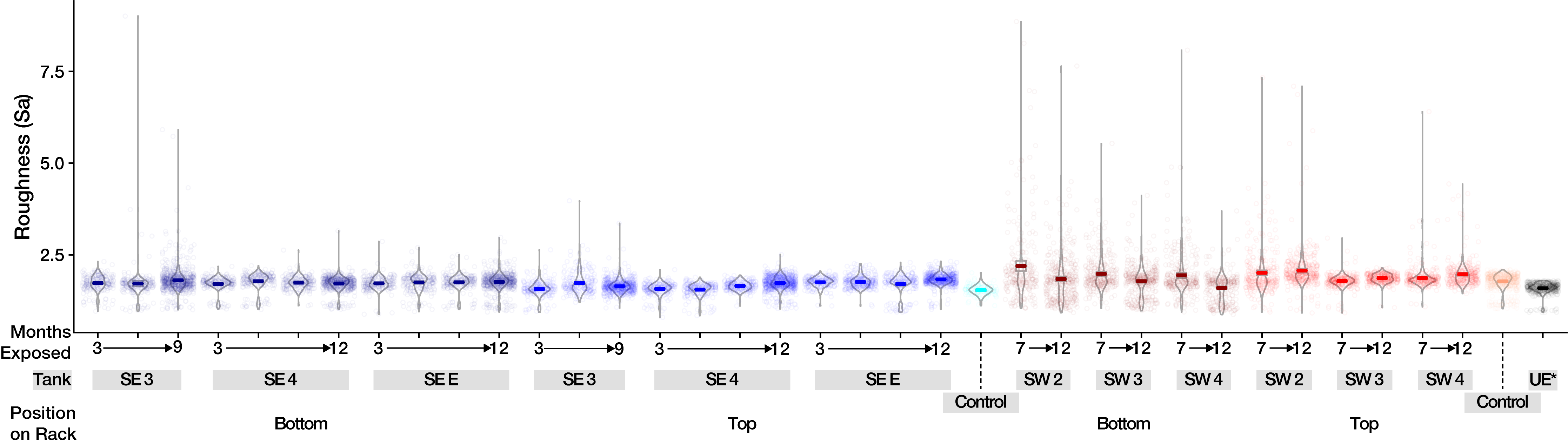

Supplement: FIGURE S8 — Roughness (Sa) values of uncoated steel witness coupons from SE (Blue) and SW (Red). [file Image_8.TIF]

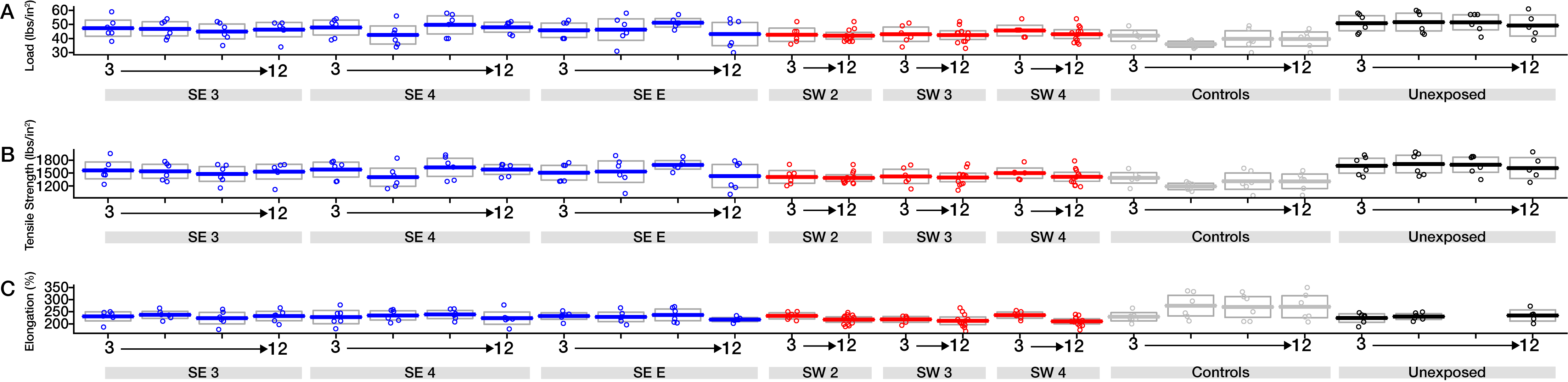

Supplement: FIGURE S9 — O-ring measurements of load (A), tensile strength (B), and elongation (C) after exposure to fuels at SE (Blue) and SW (Red). Controls exposed to SE fuel are shown in gray, and unexposed controls are represented in black. Units for load and tensile strength given in lbs/in2, and elongation as percent from original length. [file Image_9.TIF]
